# Feasibility of evaluating COVID-19 vaccine effectiveness and variant severity through a routine health information exchange.

**DOI:** 10.23889/ijpds.v7i3.2066

**Published:** 2022-08-25

**Authors:** Andrew Boulle, Alexa Heekes, Hannah Hussey, Reshna Kassanjee, Mary-Ann Davies

**Affiliations:** 1 ICES

## Objectives

To date South Africa has experienced four distinct COVID-19 waves due to ancestral, Beta, Delta and Omnicron SARS-CoV-2 variants. We sought to answer pertinent public health questions in a timely manner as new COVID-19 variants emerge(d) using routine health service data linked through a service-facing health information exchange (HIE).

## Approach

A population cohort was defined amongst regular health service users in the Western Cape Province of South Africa based on recent utilisation of public sector services as reflected in the Provincial Health Data Centre (PHDC) which functions as a HIE. Infection, hospitalisation and mortality data were derived from routinely linked laboratory, service and national vital registration data sources. Serology done on residual specimens of patients monitored for HIV and diabetes treatment progress were linked to the PHDC, as were vaccination data from the national vaccination information system. A single linked and de-identified dataset was exported for analysis purposes.

## Results

Based on accessing services in the preceding 3 years, a cohort of 3.5 million adult patients could be enumerated and linked to co-morbidity and SARS-CoV-2 outcome data. Serology from 16,000 specimens spread across the three inter-wave periods, and vaccine data from amongst the 5 million vaccine doses given in the Province, could also be linked. Variants could be identified by wave or by PCR assay target anomalies during cross-over periods. Publishable variant severity analyses were feasible from the sub-cohort of patients with diagnosed COVID-19, and variant-specific vaccine effectiveness was assessible amongst cases, in the population cohort, and in patients with HIV. The impact of prior infection and marginal value of vaccination in those with prior infection was assessible within the serology sub-cohort.

## Conclusion/Implications

A single linked de-identified dataset derived from an operational HIE was able to quickly address critical public health questions related to COVID-19 variants in a privacy-preserving manner.

